# ADVANCING ORAL HEALTH AND ORAL HEALTH–RELATED QUALITY OF LIFE MEASURES ACROSS DIVERSE POPULATIONS

**DOI:** 10.1093/geroni/igae098.1426

**Published:** 2024-12-31

**Authors:** Bei Wu, Elisa Ghezzi, Stephen Shuman

**Affiliations:** Chair; Co-Chair; Discussant

## Abstract

This symposium demonstrates the intricate interplay between social and behavioral factors and their impact on the oral health status of older immigrant populations in the United States. It also examines the efficacy of self-reported oral health measures within diverse populations. Understanding these dynamics and valid measures to be used in these populations is crucial for developing targeted interventions and policies to address oral health disparities within these communities. The first study scrutinizes oral health trends among older adults based on their immigration status in the United States, leveraging data from the National Health and Nutrition Survey. Findings reveal an overall decline in self-reported fair/poor oral health from 1999 to 2018, yet significant oral health disparities persist, notably among non-US citizens. The second study investigates the correlation between caregiving circumstances, care recipient characteristics, and oral hygiene assistance among dementia caregivers within Chinese American communities. Insights stem from a pilot study conducted in New York City. The third study delves into the relationship between support from adult children and perceived oral health among both foreign-born and U.S.-born Chinese Americans. This study provides evidence for resilience pathways that connect social support from children to older immigrants’ oral health. Lastly, based on a systematic review, the fourth study unpacks the construct of oral health-related quality of life (OHRQoL) within existing measures for adults, assessing their psychometric properties. Six distinct subdomains emerge, including pain/discomfort, oral diseases and disorders, functional limitations, oral hygiene, oral hygiene behavior, and psychosocial impact.

